# Botox Injection Combined With Partial Arytenoidectomy: A Novel Approach for Treatment of Episodic Laryngeal Breathing Disorders

**DOI:** 10.1002/wjo2.70171

**Published:** 2026-11-02

**Authors:** Zhou Zhou, Jia‐Hui Wu, Pei‐Kai Yan, Xiao‐Xiao Li, Jing Kang, Ping‐Jiang Ge

**Affiliations:** ^1^ Department of Otolaryngology, Shenzhen People's Hospital (The Second Clinical Medical College Jinan University; The First Affiliated Hospital of Southern University of Science and Technology) Guangdong China; ^2^ Department of Otolaryngology, Guangdong Provincial People's Hospital (Guangdong Academy of Medical Sciences) Southern Medical University Guangdong China

**Keywords:** arytenoidectomy, botulinum toxin, dyspnea, episodic laryngeal breathing disorders, laryngospasm, vocal cord dysfunction

## Abstract

**Objective:**

The aim of this study was to develop effective treatments for episodic laryngeal breathing disorders (ELBD), a series of diverse obstructive respiratory disorders with an unknown pathogenesis. The diseases include paradoxical vocal fold motion/movement disorder (PVFMD), vocal cord dysfunction (VCD), irritable larynx syndrome (ILS), exercise‐induced laryngeal obstruction, and laryngospasm.

**Methods:**

Five patients with ELBD were involved in this study (60–81 years, average of 70 years), including three men and two women, who visited the otolaryngology outpatient department suffering from dyspnea several times. Bilateral vocal fold paralysis and asthma were excluded. An injection of initial botulinum toxin into the vocal cords was treated for each patient. If recurrence were to take place within 3 months after injection, a vocal cord process resection would be performed. The visual scale of perception was used for assessing preoperative and postoperative dyspnea.

**Results:**

All patients experienced symptoms of recurring dyspnea for a duration of 1–120 months. The average follow‐up duration was 14 months (6–24 months) after treatment. Dyspnea was relieved in two patients after the Botox injection alone. Three patients found relief after unilateral vocal cord process resection. All patients showed a significant reduction in postoperative dyspnea scale in VAS score.

**Conclusion:**

This study preliminarily explores the efficacy of combined Botox injection and partial arytenoidectomy as a therapeutic option for ELBD.

## 1 Introduction

Episodic laryngeal breathing disorders (ELBDs) represent a series of diverse obstructive respiratory disorders with unidentified reasons. Recurrent periodic and acute breathlessness or suffocation are present in patients. ELBD encompasses a series of disorders, including paradoxical vocal fold movement disorder (PVFMD), vocal fold dysfunction (VCD), irritable laryngeal syndrome (ILS), exercise‐induced laryngeal obstruction(EILO), laryngospasm, Munchausen stridor(MS), among others, of which hint functional laryngeal respiratory pathologies. Breathlessness, shortness of breath, laryngeal stridor, laryngeal muscle tension, dysphonia, swallowing difficulty, and cough due to irritation‐induced vocal fold dysfunction characterized these disorders. The severity of airway obstruction relies on the duration and extent of improper glottic closure. However, it is challenging to distinguish ELBD from other lesions, such as vocal cord paralysis, laryngotracheal inflammatory tumor, and laryngotracheomalacia. The causative factors remain unknown, but there is a possible link to reflux and psychological factors [1].

Previous research suggests that nearly 80% of patients diagnosed with ELBD are initially misdiagnosed, often for extended periods (an average of 4.8 years) [2]. For instance, ELBD is commonly misdiagnosed as refractory asthma, leading to unnecessary and prolonged treatment with high‐dose inhaled corticosteroids, which could result in iatrogenic conditions like Cushing's disease, obesity, osteoporosis, diabetes, and hypertension [3, 4]. Moreover, intubation procedures or tracheotomies may be administered erroneously [5]. Despite over 200 case reports on suspected ELBD published over the past 140 years [6], a definitive consensus on clinical diagnostic criteria is lacking. Owing to variations in symptom manifestation, laryngoscopic findings, and triggering stimuli among individuals, ELBD diagnosis is usually undefined [7, 8, 9, 10]. Treatment strategies involve rest and avoidance of irritants, psychotherapy, voice training, acid suppression therapy, and managing complications such as laryngeal edema or asthma. Some patients alleviated their breathlessness through behavioral training and trigger removal. However, in severe cases, conventional methods were proved ineffective. Considering this lack of treatments, alternative approaches like injections or surgery to enlarge the glottis are being explored with variable success in this study.

## 2 Materials and Methods

### 2.1 Participants

This study was conducted in Guangdong Provincial People's Hospital, Otolaryngology and Head & Neck Surgery Department, From January 2017 to April 2023. A detailed version of the protocol has been approved by the Guangdong Provincial People's Hospital ethics committee [Approval number GDREC2017291H(R1)]. The diagnosis was determined through a laryngoscope examination and respiratory disease history and examination. Patients were excluded from the study if they had abnormal larynx findings, such as masses, vocal fold paralysis, or laryngeal or subglottic stenosis. Additionally, chronic obstructive pulmonary disease (COPD), asthma, sarcoidosis, bronchiectasis, etc., that were likely causing the dyspnea symptoms, as diagnosed by a pulmonologist, were conditions of exclusion. Finally, patients with other medical conditions influencing breathing, like diaphragmatic paralysis, were excluded from the study. Five patients with ELBD were involved in this study.

### 2.2 Botulinum Neurotoxin Injection

After local anesthesia, the patients were seated and injected with botulinum toxin under a fiber‐optic laryngoscopy visual. The unilateral or bilateral thyroarytenoid muscle and lateral cricoarytenoid muscle were injected with botulinum toxin(BOTOX, ALLERGAN Inc) through the cricothyroid membrane. Each vocal cord was administered with a dosage of 5U of botulinum toxin. If there's a recurrence of acute respiration within 3 months after injection, we proceed with an arytenoidectomy.

### 2.3 Surgical Technique

Under general anesthesia, the viable endoscope was used to achieve ideal glottic exposure, and the surgical side was selected based on adequate visualization under suspension laryngoscopy. The operation was initiated by making a transverse cut into the unilateral vocal cord. The location of this laceration was optimally the apex of the arytenoid's vocal process. The CO_2_ laser beam was set on the continuous mode at 4 watts. The inner and anterior portions of the arytenoid cartilage and mucosa covered were removed by CO_2_ laser. Figure 1 shows the extent of the wound after resection of the right side vocal process and partial arytenoid cartilage under laryngoscopy.

**Figure 1:**
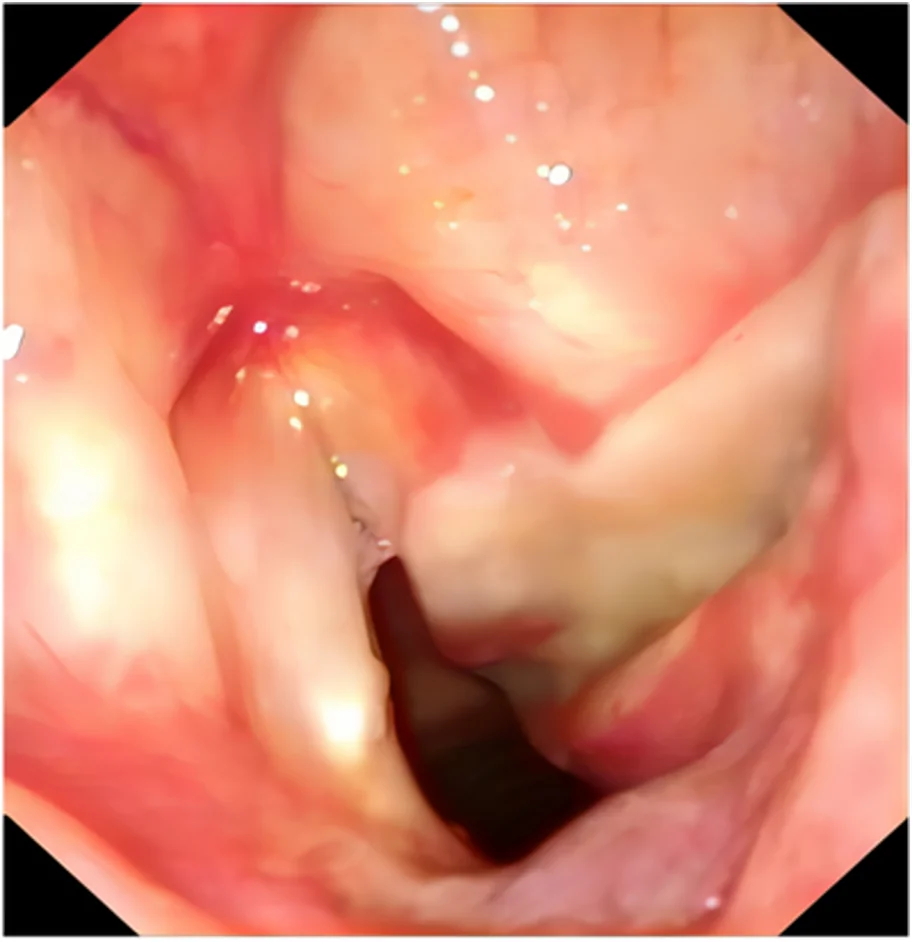
Postoperative laryngoscopy picture of partial arytenoidectomy.

### 2.4 Evaluation

The patient's dyspnea was assessed using the mMRC (modified Medical Research Council) dyspnea scale [11]at pre‐operation and post‐operation by an experienced clinician. Additionally, the patients gauged the degree of preoperative and postoperative dyspnea via the self‐perception score visual scale. This self‐assessment tool required patients to evaluate their breathing capability on a scale of 0 to 10. Herein, 0 signified an absence of dyspnea, indicating the best possible condition, while a rating of 10 indicated the maximum intensity of dyspnea, signifying the worst possible condition. The median postoperative follow‐up period was 14 months, with a range of 6 to 24 months. These five patients were all aged and had low education levels. It was difficult to be trained for the examination of voice and communication, so only the most basic and simple subjective assessment methods of voice and swallowing were given.

## 3. Result

3

Five patients were involved in this study: three males and two females (60 to 81 years, averaging 70 years). The maximum duration of symptoms was ten years, and 1 month at the minimum symptom history. The five patients all presented with dyspnea, with episodes occurring from days to weeks apart, then recuperating normally without episodic symptoms. Notably, four patient presented clear triggers by their bouts of choking. Patients 2 and 4 experienced dyspnea following physical exertion and episodes of fatigue, while Patient 3 reported a subsequent dyspnea with exposure to triggers. Patient 5, in contrast, demonstrated a susceptibility to dyspnea after stress. Patient 1 did not have an obvious trigger, potentially due to the short time history (only 1 month) or the lack of identifiable triggers. It is worth noting that patients 2 and 4 also exhibited hoarseness.

Both brain and lung lesions or accidents were conclusively excluded from all five patients, and a laryngoscopy conclusively ruled out bilateral vocal cord paralysis. One patient's medical history reported Intensive Care Unit(ICU) intubation history due to airway choking, which improved completely after tube withdrawal. None presented any significant gastroesophageal reflux symptoms in their medical history.

After administering a simple vocal cord injection of botulinum toxin, dyspnea symptoms were relieved in patients 1 and 5. The rest of the patients recurred dyspnea symptoms from 10 days to 2 months after injection. They required unilateral partial arytenoidectomy to relieve dyspnea. All patients observed a significant decrease in the degree of dyspnea after injection and operationvia visual analog scale (VAS) scores, even if some patients' symptom was triggered. The demographic details and symptoms of all patients are outlined in Table 1. Table 2 presents an evaluation of dyspnea and the outcomes of laryngoscopic examinations. None of the patients encountered major postoperative complications, including dysphonia or swallowing disorder. Each patient had no communication impairment.

**Table 1:** Demographics, symptoms, and treatments of the patients.

Items	Age	Sex	Duration (months)	Correlative cause	Hoarseness(with or without)	Frequency and duration of episodes	Treatment strategies
Patient 1	81	F	1	unclear	without	Approximately once per week, each session lasting 5 min.	5U Botox injection, unilateral
Patient 2	72	M	120	physical activities	with	The frequency of episodes is variable, with each episode lasting approximately 30 min.	10U Botox injection, bilateral, partial arytenoidectomy
Patient 3	60	F	12	catch a cold/cough	without	Persistent dyspnea	5U Botox injection, unilateral, partial arytenoidectomy
Patient 4	66	M	12	overworked, physical activities	with	Six times per year, each episode lasting more than half an hour	5U Botox injection, unilateral, partial arytenoidectomy
Patient 5	67	M	6	stress	without	The frequency ranges from several days to several weeks per episode, with each episode lasting dozens of minutes.	5U Botox injection, unilateral

Abbreviations: F, female; M, male.

**Table 2:** Dyspnea assessments and laryngoscopic findings of patients.

Items	Patient 1	Patient 2	Patient 3	Patient 4	Patient 5
Pre‐injection mMRC	2	3	2‐3	3	2
Post‐injection mMRC	0	Dyspnea recurs 90 days after injection	Dyspnea recurs 120 days after injection	Dyspnea recurs 10 days after injection	0
Preop mMRC	/	2–3	2–3	2–3	/
Postop mMRC	/	0	0	0	/
Pre‐injection VAS	6	9	8	9	6
Post‐injection VAS	0	Dyspnea recurs 90 days after injection	Dyspnea recurs 120 days after injection	Dyspnea recurs 10 days after injection	0
Preop VAS	/	7	8	8	/
Postop VAS	/	0	2	0	/
Laryngoscopic findings(during inspiration)	Vocal fold adduction	Vocal fold adduction	Vocal fold adduction	Vocal fold adduction	Not obviously

*Note:* The evaluation time point for post‐injection is 3 months, while that for Postop is 6 months postoperatively.

## 4 Discussion

Regarding managing ELBDs, an unanimous agreement on the appropriate surgical approach remains to be discovered. Thus, the objective of this study was to delineate the method we employed in the treatment of ELBD and to share our findings.

### 4.1 Refractory ELBD

ELBD encompasses a broad spectrum of conditions. The classic symptoms of this disorder typically include frequent, sudden attacks of dyspnea and acute respiratory distress [1]. Irritable Larynx Syndrome (ILS) is exhibited in some patients who report variations of laryngospasms, with or without voice changes, generally as a reaction to either chronic coughing or isolated coughing fits [12]. According to the report of Paroxysmal Vocal Fold Movement Disorder (PVFMD) and vocal cord dysfunction (VCD), the true vocal cords abnormally adduct toward the midline when inhaling. If the adduction occurs only during inhalation, it falls under PVFMD, while concurrent inhalation and exhalation adductions would categorize it as VCD [7]. Nonetheless, some studies indicated that in some cases, this inappropriate movement of the vocal cords is not observed precisely at the time of the laryngoscopy [13]. In this study, upon video stroboscopic examination, we observed four patients' vocal cords adducting or moving toward the midline during the inspiration phase, not during the duration of the attack. However, in one patient, the adduction was not visible.

Diagnostic tests are needed to exclude pulmonary diseases, such as asthma and acute inflammation. Sometimes, severe schizophrenia may cause pseudo‐disease [14]. In this study, one patient's medical history included mental anxiety disorder, without schizophrenia. All patients were never diagnosed with acute pulmonary disease or cardiac accident by pulmonologists and cardiologists.

The patients' diverse conditions are spontaneously resolving the recurrent dyspnea. One must be careful to avoid triggering and causing paradoxical vocal fold motion/movement disorder (PVFMD), vocal cord dysfunction (VCD), exercise‐induced laryngeal obstruction (EILO), or laryngospasm. Instead, these symptoms more favorably align with ELBD diagnosis.

All five patients receiving this sequential intervention were severe refractory cases, with detailed eligibility thresholds specified as follows: Botulinum toxin injection was indicated for patients with persistent episodic attacks after failure of conservative behavioral respiratory training, featuring an attack frequency of more than six episodes per year and each unrelieved attack lasting longer than 5 min. Subsequent partial arytenoidectomy was indicated for individuals who failed botulinum toxin injection, with no obvious improvement in either attack frequency or severity after pharmacotherapy.

### 4.2 Treatment Strategies

Until now, no effective surgical treatments have been applied in the therapy of ELBD [1]. Before requiring injection and surgery treatments in our clinic, these patients sought multiple medical consultations. They underwent reflux treatments and health education interventions, where they learned techniques for calm breathing and relaxation. However, their recurrent dyspnea symptoms persisted, yielding little relief despite various treatments. Experiencing unwanted side effects and disillusionment by previous, ineffective treatments that resulted from erroneous diagnoses.

For patients with refractory ELBD, a sequential therapeutic regimen was adopted in the present study. Botulinum toxin injection was selected as first‐line management, given its proven therapeutic efficacy for a broad spectrum of laryngeal diseases [15]. Particularly for refractory paradoxical vocal fold motion disorder (PVFMD), botulinum toxin injection is regarded as the primary therapeutic option when alternative conservative treatments prove ineffective [16].

In four patients presenting with severe dysphonia, an initial unilateral injection of 5 units botulinum toxin was administered. The second patient developed progressively worsening dyspnea with elevated mMRC scores after single‐side injection and subsequently received bilateral injection at a total dose of 10 units for more intensive intervention. While all patients achieved transient symptomatic relief shortly after injection, three cases suffered recurrent dyspnea within 60 days. As previous literatures [17] have verified that the therapeutic effect of botulinum toxin lasts approximately 3 to 4 months, partial arytenoidectomy was performed for patients with unsatisfactory symptom control following toxin injection.

Tracheotomy is a reliable emergency salvage procedure for critical dyspnea; however, partial arytenoidectomy was prioritized in our cohort to alleviate respiratory distress and avoid long‐term adverse complications related to tracheostomy. Achieving an optimal balance between satisfactory airway patency and preserved vocal function is a core principle guiding surgical selection [18]. A variety of surgical techniques have been developed to expand the glottic airway in patients with bilateral vocal fold paralysis, including posterior cordectomy combined with arytenoidectomy, total arytenoidectomy, and partial arytenoidectomy [19]. Cumulative clinical consensus indicates that partial arytenoidectomy is capable of establishing sufficient glottic caliber while conserving acceptable vocal quality and lowering the risk of postoperative aspiration [20, 21].

### 4.3 Follow‐up Arrangements

We stratified follow‐up assessments into short‐term and long‐term schedules based on the pharmacological characteristics of botulinum toxin. Short‐term follow‐up was arranged at postoperative month 3, matching the average effective duration of the injectable agent. Recurrent dyspnea within 3 months was defined as treatment failure of isolated botulinum toxin injection, which served as the cutoff criterion to judge the efficacy of pharmacotherapy. It should be clarified that not every single recurrent episode within three‑months automatically triggers surgical intervention. Surgical indication was established when patients still suffered from persistent severe symptoms that substantially compromised respiratory function and quality of life, even if a certain reduction in attack frequency had been achieved. Increasing botulinum toxin dosage may represent a potential strategy to optimize therapeutic response in such scenarios. Nevertheless, given the limited sample size of the present case series, we could not draw definitive conclusions concerning dosage‐efficacy correlation. All long‐term evaluations were conducted no earlier than 6 months post‐injection to eliminate the confounding influence of residual botulinum toxin activity. Clinical outcomes assessed after complete metabolic clearance of the drug verified that postoperative symptom improvements were mainly attributed to surgical resection, instead of persistent pharmacological effects of botulinum toxin or synergistic benefits from combined therapy.

Episodic dyspnea constituted the dominant clinical complaint among our subjects, whereas vocal impairment was not a primary presenting symptom. The majority of enrolled elderly patients denied subjective swallowing discomfort. Detailed pre‐ and post‐treatment interviews focusing on voice and deglutition, together with routine clinical examinations, revealed no notable abnormalities in vocal or swallowing function across the cohort. For this reason, standardized objective grading systems including Grade‐Roughness‐Breathiness‐Asthenia‐Strain (GRBAS) scale [22] and Functional Oral Intake Scale(FOIS) were not implemented routinely. After partial arytenoidectomy, none of the enrolled patients experienced recurrent dyspnea, symptomatic vocal deterioration or postoperative aspiration events.

## 5 Conclusion

Based on the evaluation of this limited case series, combined botulinum toxin injection (with or without partial arytenoidectomy) can markedly alleviate dyspnea, reduce seizure frequency, or lessen attack severity in a subset of patients with refractory ELBD.

## Author Contributions

**Zhou Zhou:** methodology, data collection, and draft writing. **Jia‐Hui Wu, Pei‐Kai Yan** and **Xiao‐Xiao Li:** data collection and formal analysis. **Jing Kang:** review writing and revision, and editing the final draft. **Ping‐Jiang Ge:** methodology, idea formulation, and final revision.

## Ethics Statement

This study was performed in line with the principles of the Declaration of Helsinki. Approval was granted by the Guangdong Provincial People's Hospital ethics committee [Approval number GDREC2017291H(R1)].

## Conflicts of Interest

The authors declare no conflicts of interest.

## Supporting information


Supporting File 1

Supporting File 2

## Data Availability

The data that supports the findings of this study are available in the supporting material of this article The authors affirm that human research participants provided informed consent for publication of the image in Figure 1. Informed consent was obtained from all individual participants included in the study.
